# Advancements on the Multifaceted Roles of Sphingolipids in Hematological Malignancies

**DOI:** 10.3390/ijms232112745

**Published:** 2022-10-22

**Authors:** Yasharah Raza, Jane Atallah, Chiara Luberto

**Affiliations:** 1 Department of Pharmacological Sciences, Molecular and Cellular Pharmacology, Stony Brook University, Stony Brook, NY 11794, USA; 2 Stony Brook Cancer Center, Stony Brook University Hospital, Stony Brook, NY 11794, USA; 3 Department of Physiology and Biophysics, Stony Brook University, Stony Brook, NY 11794, USA

**Keywords:** sphingolipids, hematological malignancies, leukemia, myeloma, lymphoma, ceramide, sphingosine-1-phosphate, sphingolipid metabolism, acid ceramidase, sphingosine kinase-1

## Abstract

Dysregulation of sphingolipid metabolism plays a complex role in hematological malignancies, beginning with the first historical link between sphingolipids and apoptosis discovered in HL-60 leukemic cells. Numerous manuscripts have reviewed the field including the early discoveries that jumpstarted the studies. Many studies discussed here support a role for sphingolipids, such as ceramide, in combinatorial therapeutic regimens to enhance anti-leukemic effects and reduce resistance to standard therapies. Additionally, inhibitors of specific nodes of the sphingolipid pathway, such as sphingosine kinase inhibitors, significantly reduce leukemic cell survival in various types of leukemias. Acid ceramidase inhibitors have also shown promising results in acute myeloid leukemia. As the field moves rapidly, here we aim to expand the body of literature discussed in previously published reviews by focusing on advances reported in the latter part of the last decade.

## 1. Introduction

The investigations into the bioactive functions of sphingolipids (SLs) in the regulation of critical cellular functions, such as apoptosis, were spearheaded by the ground-breaking finding that sphingosine regulates apoptosis in HL-60 pro-myelocytic leukemic cells [1]. This relationship not only established a novel function for SL metabolism in addition to the cellular structural role of some of its molecules, but permanently linked SL metabolism with hematological malignancies. This has provided the framework for an expanding body of research and has led to many new discoveries on SLs in hematological malignancies. SLs have been shown to have roles in cellular differentiation, senescence, proliferation, and more, in a variety of normal and pathological conditions, including various hematological malignancies such as leukemias, lymphomas, and myelomas [2,3,4,5]. This area of research continues to grow, providing new insights on mechanisms and functions of SLs in various disease states, as well as novel therapeutic targets in hematological malignancies. The aim of this review is to expand on the body of literature in this field and provide an update on newly reported associations between SLs and hematological malignancies over the latter half of the last decade. 

### A Brief Introduction to SL Metabolism and Signaling

SLs comprise a heterogeneous group of several structural and bioactive lipids that play important roles in different cellular functions including growth, senescence, apoptosis, adhesion, migration, cell trafficking, angiogenesis, and inflammation [2,6]. Importantly, dysregulation of SL degradation is the cause of over 40 inherited lysosomal storage diseases, such as Gaucher, Nieman–Pick, or Farber disease to mention a few (for a comprehensive review, refer to [7,8]). More recently, additional disorders have also been connected to genetic alteration of other components of the SL pathway, such as mutations in genes regulating de novo SL synthesis (i.e., Serine palmitoyltransferase long chain base subunit 1 or one of its regulators, *ORMDL3*) and others involved in the catabolism of SLs (i.e., Alkaline Ceramidase 3 and Sphingosine-1-Phosphate Lyase) [8,9,10,11]. It is safe to say that alterations in SL metabolism (either genetic or acquired) have been associated with pathological conditions in nearly all systems of the body, causing neuronal, respiratory, glomerular, cardiovascular, and epidermally related disorders. The reader is referred to the following reviews to begin gaining a wider scope on the plethora of disease states impacted by SL dysregulation [7,12,13,14,15,16,17,18,19,20,21,22,23].

**Figure 1:**
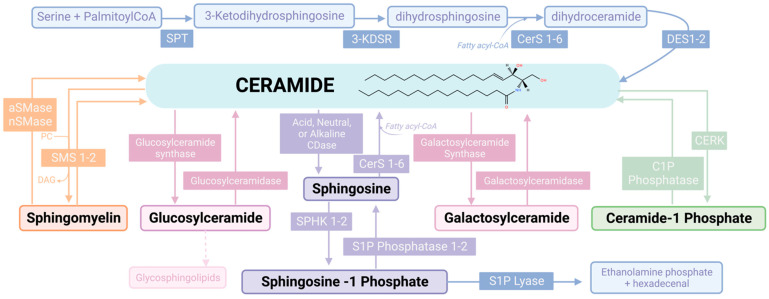
A simplified overview of the sphingolipid metabolic pathway. Central to sphingolipid metabolism is ceramide synthesis and regulation. The pathway in light blue above ceramide represents the de novo pathway of ceramide synthesis. The other pathways branching from ceramide represent other means of synthesis and degradation of ceramide and of other sphingolipid molecules. The figure was created using BioRender.com (accessed on 21 October 2022). C16:0-ceramide structure from www.lipidmaps.org (accessed on 21 October 2022). SPT: serine palmitoyltransferase; 3-KDSR: 3-ketodihydrosphingosine reductase; CerS: ceramide synthase; DES: dihydroceramide delta(4)-desaturase; aSMase: acid sphingomyelinase; nSMase: neutral sphingomyelinase; SMS: sphingomyelin synthase; DAG: diacylglycerol; PC: phosphatidylcholine; CDase: ceramidase; SPHK: sphingosine kinase; S1P: sphingosine-1-phosphate; C1P: ceramide-1-phosphate; CERK: ceramide kinase.

The SL pathway is characterized by a complex network of interconnected reactions distributed among most cellular compartments (Figure 1). 

SL synthesis begins in the endoplasmic reticulum with the condensation, for the most part, of a molecule of serine and one of palmitoyl-CoA to form 3-ketodihydrosphingosine through the action of an enzymatic complex referred to as the Serine Palmitoyl Transferase (SPT) [24,25]. 3-ketodihydrosphingosine is reduced to dihydrosphingosine (dhSph) by the 3-ketodihydrosphingosine reductase (3-KDSR) [26,27]. DhSph is either phosphorylated to dhSph-1P by one of two sphingosine kinases (SPHK1 or 2) [28] or it is converted into dihydroCeramide (dhCer) with the addition of a fatty acyl group through the action of the ceramide synthases (CerS 1–6) [29]. DhCer is then desaturated to form ceramide via the action of the dihydroceramide Δ4-desaturase (DES) enzyme. Ceramide can in turn be metabolized into several different molecules [29]. Ceramide, in fact, can serve as the building block for the synthesis of more complex SLs with different head groups: 1. sphingomyelin (SM) via the action of sphingomyelin synthases (SMS1,2) [30,31,32], 2. ethanolamine phosphoceramide by the sphingomyelin synthase related protein (SMSr) [32], 3. glycosphingolipids, starting with the initial conversion into glucosyl- or galactosyl-ceramide by the action of glucosylceramide or galactosylceramide synthase, respectively [33,34], 4. ceramide-1-phosphate by ceramide kinase [35,36], and 5. acylceramide via the activity of the long-chain-fatty-acyl-CoA ligase 5 (ACSL5) first and of the diacylglycerol O-Acyltransferase 2 (DGAT2) [37] to follow. 

Most complex SLs are broken down by specific hydrolytic enzymes. For instance, SM is hydrolyzed back to ceramide by one of five different sphingomyelinases (acid, alkaline, and three neutral SMases) [38,39,40]; hexosylceramides are also metabolized by either glucosylceramidase beta (GBA1-3) or by the galactosylceramidase [41,42]. Ceramide itself can be hydrolyzed into sphingosine (Sph) by the action of several ceramidases (acid, neutral, and three alkaline ceramidases) [43] and Sph can in turn be phosphorylated by the SPHK1 and 2 to form sphingosine-1-phosphate (S1P) [44,45]. 

SLs are a heterogeneous group of molecules not only because they comprise different SL classes as briefly discussed above, but also because each class counts different molecular species. This intra-class heterogeneity is dictated by: 1. the existence of multiple isoforms of the CerS biosynthetic enzymes (CerS1-6), each with different fatty acyl preference [46], and 2. different combinations of enzyme subunits forming the SPT which allow the use of alternative amino acids and acyl-CoAs as substrates (in addition to serine and palmitoyl-CoA) [47,48]. As a result, many different molecular species of ceramides and downstream complex SLs are formed, diverse either in their sphingoid backbone or in the fatty acyl chain they carry. 

Most importantly, SL heterogeneity (within and across SL classes) is the basis for the diverse array of biological functions mediated by this pathway. For instance, while ceramide has been historically considered a pro-apoptotic molecule, more recent advances have unveiled a more complex picture whereby the same ceramide molecular specie or different molecular species can exert different, even opposing functions depending on the cellular context. An example of the multifaceted aspects of ceramide signaling is C16:0-ceramide. While the accumulation of C16:0-ceramide following engagement of the T-cell receptor has been associated with cell death of B-cells [49], accumulation of the same C16:0-ceramide via activation of CerS6 in head and neck cancer cells promotes proliferation and tumor growth in severe combined immunodeficiency mice [50,51]. Additionally, while radiation-mediated activation of ceramide synthases CerS5 and CerS6 induces cell death in human cervical cancer (HeLa) cells, CerS2 and its product, C24:0-ceramide, confer resistance to radiation in the same cell line [52]. Hence, each ceramide specie must be considered an individual bioactive molecule with specific biologies depending on the inducer and cell environment.

Similarly, Sph is generally associated with negative effects on cell proliferation and mostly promotes cell death [53]; however, this depends on the extent of its formation and how this contributes to further downstream metabolism. For instance, while production of Sph triggered by sustained activation of ACER2 following DNA damage causes caspase-dependent cell death, production of relatively lower levels of Sph can fuel S1P formation [54], which instead is mostly associated with positive effects on cell proliferation and survival [55,56]. Ultimately, it is the relative balance of the various SL enzymatic activities/SL levels that determines the final biological effects [57].

Several molecular targets for ceramide, Sph, and S1P have been identified and invoked to mediate their bioactive functions. Among the reported ceramide direct targets were first proteins whose enzymatic activity was impacted by ceramide in vitro, such as the protein kinases KSR and PKCζ and protein phosphatases PP1 and PP2a [58,59,60,61]. Subsequently, a few of these proposed direct targets were confirmed by different pull-down strategies (PP1 and PP2a regulatory subunits, PKCζ, Raf-1), while additional ones were identified, such as TP53, LC3B-II, tubulin, and VDACs [62,63,64,65,66,67,68,69], to name a few. Of note, the wide range of ceramide direct targets and ceramide-mediated structural changes in membranes support the variety of its biological functions, including cytokine secretion, vesicle secretion, autophagy, cell death, differentiation, senescence, and modulation of adhesion and migration [23,70,71,72,73,74,75,76].

Fewer proximal targets have been linked to Sph; this is likely due to the rapid metabolic conversion of Sph into ceramide and S1P in cells and the challenge to “trap” Sph interactors [77]. Historically, the first observation indicative of a signaling function for SLs came from inhibition of Protein Kinase C (PKC) by Sph in vitro [78]. In cells, Sph accumulation has been linked to the inhibition of Akt, Erk, and PKC or activation of protein kinase A, Jun kinase/p38, or PP1 and to dephosphorylation of Rb [53,79,80,81,82,83,84,85]. Incidentally, many of these sphingosine targets were identified in blood cancer cell lines. Biologically, these changes were connected to mitochondrial damage, caspase activation, autophagy, and/or cell death. More recently, transient elevation of Sph in the lysosomes (using caged Sph analogs) led to a transient release of calcium from these organelles, due to enhanced activity of the two-pore channel 1 (TPC1) in endosomes and lysosomes [86].

S1P acts both extracellularly and intracellularly. Extracellularly, S1P is recognized by 5 G-protein coupled receptors (named S1P Receptor 1-5, S1PR1-5) [87], whose level of expression on the plasma membrane depends on the cell type. The specific pattern of engagement of S1PRs defined by the cellular context and the specific combination of downstream mediators results in various biologies, including cell growth and survival, migration, invasion, and inflammation. More recently, intracellular targets of S1P have also been identified, such as TRAF2, PPARγ, PHB2, HDACs, and telomerase [56,88,89,90,91]. The direct interaction of S1P with such targets ultimately leads to modulation of inflammatory genes, angiogenesis, mitochondrial respiration, gene transcription, and tumor growth, respectively.

Structural functions of SM and glycoSLs in the membranes have also been linked to the regulation of signaling. For instance, SM and glycoSLs have long been proposed to be essential components of membrane lipid microdomains. These microdomains, often referred to as lipid rafts, are thought to promote select receptor-mediated signaling [92,93]. Hence, SM, by contributing to the homeostasis of these domains, indirectly modulates the activity of some membrane receptors [94,95,96,97].

In addition to its structural role within lipid rafts and as a direct product of ceramide metabolism, relevant to this review is the fact that the glycosphingolipid glucosylceramide (GluCer) has been found to regulate levels of P-glycoprotein (P-gp/*MDR1*). P-gp is an important membrane efflux pump that extrudes xenobiotics. Particularly, it has been reported that increased synthesis of GluCer promotes expression of P-gp [98], while downregulation of *UGCG* (the gene encoding glucosylceramide synthase—GCS) reduces P-gp levels and sensitizes drug-resistant breast and leukemic cells to chemotherapy [99,100]. The reverse relationship was also demonstrated, with inhibition of P-gp reducing GluCer synthesis also in leukemic cells [101,102]. This effect was explained by the feedforward effects on GluCer synthesis exerted by the reported transport of GluCer via P-gp from the cytosolic side of the Golgi (where GluCer is synthesized) to the luminal side [103]. The link between GluCer and P-gp together with the reported role of GCS as a ceramide “detoxifying” enzyme has implicated glycoSL synthesis in multidrug resistance.

From this very brief overview of the most extensively studied bioactive nodes within the SL metabolism and most relevant to our theme, it is clear as their actions affect a variety of downstream signals and functions. Within the SL pathway, there are additional signaling molecules that are equally relevant in many contexts, and possibly even in hematological malignancies as we continue to learn and explore. Therefore, we strongly encourage readers, particularly the less experienced in SLs, to refer to the referenced comprehensive reviews to build a more thorough understanding of the many nuances of SL-mediated signaling and (patho)-physiology [2,4,57,104,105,106,107,108,109]. 

Several lines of evidence have supported a link between SLs and cancer, documenting both tumor intrinsic effects and effects on the tumor microenvironment. For instance, the accumulation of cellular ceramide has been linked to the cytotoxic effects of chemotherapy in cancer cells [110,111] while altered expression of several enzymes of SL metabolism has been implicated in drug resistance [112]. As a corollary, inhibition of the enzymes that metabolize ceramide can also sensitize cancer cells to chemotherapy [113,114,115,116]. More recently, SLs have also been shown to play important functions in the tumor microenvironment (including modulation of the anticancer immune response) and either boosting or curbing tumorigenesis [117,118,119,120]. 

Targeting SLs may also have potential therapeutic applications for hematological malignancies, including various leukemias [121]. However, the molecular heterogeneity, even within the same SL class, the interconnected nature of SL metabolism, and the often opposing functions of different SLs must be taken into account while considering potential therapeutic applications. As briefly mentioned, SLs have long been implicated in hematological malignancies. Thorough reviews on the roles of SLs in various hematological malignancies have been published previously [121,122,123,124,125,126,127,128] and we strongly encourage all readers to peruse these to obtain a robust foundation for the updates we will be presenting herein. 

## 2. SLs in Hematological Malignancies

While the discovery of the roles of SLs as bioactive molecules in hematological malignancies dates back to the 1980s, experimental evidence of their complex roles in normal hematopoiesis has been uncovered more recently [129]. As hematological malignancies often rise due to aberrations in the process of normal hematopoiesis and hematopoietic differentiation, these recent advances linking SLs and lineage commitment provide a conceptual framework for a better understanding of the various effects of SLs also in hematological malignancies.

The following sections will summarize the most recent findings on SLs in hematological malignancies in the context of the most critical previous discoveries. The sections are organized broadly by the classification of hematological malignancies, namely, leukemias, lymphomas, and myelomas. Specific subtypes are addressed within each section where relevant. 

### 2.1. Leukemia

Leukemia is one of the first experimental model systems in which bioactive SL functions were identified. In fact, the discovery that Sph regulated apoptosis in pro-myelocytic HL-60 leukemic cells marked one of the first links to SL involvement in hematological malignancies [1]. Since then, a plethora of findings substantiated the bioactive roles of different SLs in hematological malignancies. 

Recently, an inhibitory screen for SL genes using CRISPR/Cas9 found that expression of 3-KDSR was particularly relevant in leukemic cells compared to other cancer cell types [130]. In fact, in acute myeloid leukemic cell lines, MV4-11 and MOLM-13, and in other leukemic cells, SL modulation by 3-KDSR is necessary to maintain the unfolded protein response (UPR) following endoplasmic reticulum stress [130]. UPR is essential for responding to the accumulation of misfolded or unfolded proteins in the ER typical of various types of leukemia and to mediate drug resistance [131,132]. Therefore, the link between KDSR and UPR may be a promising target for alleviating therapeutic resistance in these leukemias. 

In addition to the bioactive roles of SLs in leukemia, some of these lipids also serve as potential biomarkers. In a study profiling SM levels in sera from patients with different hematological malignancies, it was found that, while the total SM concentration is not different in malignant versus normal sera, the profile of the various species of SM is different [133]. Serum samples from patients with acute myeloid leukemia, acute lymphocytic leukemia, B-cell lymphoma, or myelodysplastic syndrome all had more SM species carrying even-chain fatty acids, such as C16:0-SM, C16:1-SM, and C18:0-SM, and less SM with odd-chain fatty acids such as C15:0-SM, C21:0-SM and C23:0-SM compared to normal samples. This finding purports that the ratio of different SM species may serve as a biomarker of different types of leukemia or hematological malignancies [133]. 

Several additional roles have been reported for a variety of SLs in leukemia and those roles will now be explored in the following sections. 

#### 2.1.1. Acute Myeloid Leukemia (AML) 

AML is a hematological malignancy that occurs mostly due to mutations and genetic changes that affect normal hematopoiesis and lead to an overproduction of clonal myeloid progenitors [134]. Patients with AML may have abundant aberrant myeloid cells in their bone marrow and peripheral blood, and, as the disease advances, they may present with bone marrow failure [134,135]. First-line treatments for AML include cytarabine and daunorubicin, midostaurin, venetoclax and azacitidine, or, gemtuzumab, based on individual patient diagnoses [136]. The involvement of bioactive SLs in AML is a rapidly expanding field, with initial studies exploring SPHK1 as a therapeutic target [137,138,139] and showing decreased ceramide levels associated with increased GCS and SMS activities as indicative of chemoresistance [140].

I. Prosurvival/Anti-Apoptotic Effects of SPHK1 and Ceramidases

Recent findings have explored SL involvement in proliferation, apoptosis, and resistance to apoptosis, DNA methylation, mitophagy, and chemoresistance in AML. One of the enzymes recently linked to the insurgence of erythroleukemia was SPHK1 [138]. In a model of erythroleukemia induced by overexpression of *SPI1/PU.1* in proerythroblasts, as well as in *Spi-1* overexpressing transgenic mice, *Sphk1* was found to be transcriptionally upregulated. In vitro evidence linked *Sphk1* upregulation in murine erythroleukemic cells with resistance to apoptosis as well as increased proliferation, and in vivo engraftment of *Sphk1* overexpressing proerythroblasts led to increased tumorigenicity in 8-10 week old nude mice. SPHK1 inhibition with 10 μM dimethylsphingosine reversed these effects and reduced proliferation and survival of these erythroleukemic cells in vitro [138]. These results have strengthened the evidence of SPHK1 as a newfound player in the development and survival of erythroleukemic cells and may provide a foundation for future therapeutic exploration of SPHK1 in acute erythroleukemia. 

Similarly, a role for SPHK1 in sustaining the survival of AML cells was shown in HL-60, multi-drug-resistant HL-60/VCR, and U-937 cell lines. Inhibition of SPHK1 (and SPHK2) with SKI-178 has been shown to induce apoptosis in vitro in HL-60 cells as well as to reduce leukemic burden in vivo in mice engrafted with MOLM-13 cells [141]. Further to this, the cell death caused by SPHK1 inhibition was recently shown to be mediated by degradation of the pro-survival myeloid cell leukemia-1 protein (Mcl-1) [142,143]. In this model, the use of MP-A08, an inhibitor of SPHK1 (and to a lesser extent of SPHK2) induced ceramide accumulation which in turn initiated a stress response that ultimately targeted and degraded Mcl-1 [142]. Mcl-1 is essential for AML cell survival [144], thus the link between ceramide and Mcl-1 degradation in AML is a promising novel therapeutic target to be explored for the treatment of AML. Additionally, inhibition or knockdown of SPHK1/*SPHK1* led to lower survival of primary AML blasts, while importantly, SPHK1 inhibition in normal CD34^+^ cells did not exert the same effect, confirming SPHK1 dysregulation as a player in leukemic cell survival [145]. In line with the previous study, SPHK1 inhibition by MP-A08 in MV411 AML cells was recently shown to cause ceramide accumulation and subsequent apoptosis [142]. Ceramide-mediated apoptosis was attributed to the induction of an integrated stress response based on upregulation of NOXA and protein kinase R (PKR), and activation of ATF4 [142,146,147,148]. The upregulation of NOXA was particularly interesting because NOXA is a BH3-only protein that is known to promote apoptosis through inhibition of BCL2 and Mcl-1 [149]. Capitalizing on the activation of NOXA, the combination of the SPHK1 inhibitor with venetoclax (a BH3 mimetic and inhibitor of BCL2) was shown to synergize and overcome resistance of OCI-AML3, an intrisically venetoclax-resistant cell line and in AML patient samples with mutations linked to venetoclax resistance (both in vitro and in in vivo AML PDXs) and in Leukemic Stem Cells [142]. Venetoclax is among the newer drugs approved for AML treatment in combination with low-dose cytarabine [150]; furthermore, the combination of venetoclax with hypomethylating agents, azacitidine or decitabine, earned FDA approval in 2018 for the treatment of newly diagnosed AML patients, aged 75 years or older [151]. Therefore, moving forward, the sensitization to venetoclax via inhibition of SPHK1 provides a promising strategy to maximize the antileukemic activity of venetoclax.

Whether the expression level/activity of SPHK1 is different in AML cells compared to normal stem/progenitor cells is debatable. In line with the previous studies indicating a proleukemic function for SPHK1, one report showed that *SPHK1* mRNA was highly expressed in AML cells derived from patients [145]. Additionally, in line with potentially enhanced activity of SPHK1 in AML is the reduced expression of *SKIP*, a SPHK1 inhibitor, in primary AML cells [152]. However, while SKIP has been shown to inhibit SPHK function in fibroblasts [153,154], a study in K562 Chronic Myelogenous Leukemia cells reported that SKIP acts more as a positive, rather than negative, regulator of SPHK1/S1P [155]. Moreover, it was also found that primary AML cells showed *decreased* SPHK1 and SPHK2 activity, as measured by the conversion of d17-Sph into d17-S1P [155]. Ultimately, additional studies are needed to conclusively address whether the levels of SPHK1/S1P are different in AML cells, perhaps by stratification of AML types and/or level/types of resistance. 

One of the most exciting advancements is the emergence of ceramidases as therapeutic targets for AML. Acid ceramidase (AC) has been shown to sustain survival of both primary AML cells and AML cell lines HL-60, Kasumi-1, and KG-1 [113]. Expression of *ASAH1* (gene name for AC) is significantly higher in primary AML cells compared to normal CD34^+^ cells as evidenced by both microarray analysis and TCGA RNA-Seq gene expression data [113]. Inhibition and downregulation of AC with LCL204 led to apoptosis and reduced survival of these cells both in vitro and in vivo, likely because of decreased levels of Mcl-1 [113]. Further investigation into novel drugs for AML that target SL metabolism led to the discovery of SACLAC, a ceramide analog and irreversible inhibitor of AC [156]. Consistent with the inhibition of AC, SACLAC decreased S1P and increased ceramide levels, and induced cell death of AML cells lines HL-60/VCR, THP-1, and OCI-AML2, as well as a 75% reduction in the leukemic burden of AML xenograft in NSG mice [156]. 

In addition to AC, alkaline ceramidase has also emerged as a potential novel therapeutic target in AML [157]. In fact, alkaline ceramidase 3 (ACER3) has recently been shown to support in vitro survival of AML cell lines U937, THP-1, and NB4. Similar to AC, AML cells also exhibit high *ACER3* levels, and high *ACER3* levels are associated with worse survival of AML patients [157].

II. Involvement of SPHK1, AC, and GCS in Drug Resistance

In addition to supporting the survival of AML cells, targeting SPHK1 has also been implicated in overcoming multi-drug resistance (MDR) [137]. In doxorubicin and etoposide-resistant HL-60 cells, SPHK1 activity was found to be sustained, whereas in normal non-chemoresistant HL-60 cells, SPHK1 activity was relatively low [137]. Additionally, overexpression of *SPHK1* in parent HL-60 cells protected cells from cell death upon doxorubicin or etoposide treatment [137]. 

Building upon this, recent advances have pointed to more complex changes and alterations in the sphingolipidome. AML cells resistant to the combination of current chemotherapeutics (daunorubicin and cytarabine) are characterized by a unique upregulation of AC, GCS, and SPHK1 activity, as well as increased expression of *ASAH1*, *UGCG*, and *SPHK1* [158]. In supporting the role of high levels of AC in mediating drug resistance and survival, *ASAH1* overexpression in AML cells conferred resistance to chemotherapeutics such as cytarabine, daunorubicin, and mitoxantrone [159]. Targeting AC pharmacologically with LCL204 decreased resistance to these agents [159]. Additionally, in vincristine-resistant cells, SPHK1 protein levels were also found to be elevated, along with AC and GCS protein levels [160] and a marked increases in S1P, ceramide, GluCer and SM were measured [160]. These changes were associated with altered mitochondrial bioenergetics, including increased respiration and oxygen consumption, as well as increased mitochondrial mass. Utilization of GCS and AC inhibitors, D-threo-PDMP and SACLAC further increased ceramide levels. These inhibitors in combination with metformin (an inhibitor of mitochondrial complex 1 [161]) synergized and led to more cytotoxicity than either treatment alone, exemplifying a treatment strategy that exploits the relationship between sphingolipid metabolism and mitochondrial function [160]. However, another study showed that, despite increased GCS activity in Ara-C-resistant HL-60 cells, the level of its lipid product GluCer was lower in resistant versus parental cells, possibly because of overall lower levels of ceramides in these cells [158].

III. FLT3-ITD AML

Another relevant alteration of SL metabolism, and more specifically of ceramide synthesis, is the suppression of CERS1 in the context of *FLT3-ITD* mutation [162]. *FLT3-ITD* defines an activating mutation of the Fms-like tyrosine kinase 3 (*FLT3*) known as internal tandem duplication (*ITD*), which is known to arbitrate resistance of AML, enhance survival [163], as well as promote proliferation in hematopoietic cells [164]. This specific mutation of *FLT3* is known to be present in one-third of patients with AML [165]. *FT3-ITD* AML is treated with the FLT3 inhibitor midostaurin [166] (or the recently approved Gliteritinib) [167] in combination with the typical treatment regimen of cytarabine and anthracyclines. It was found that ceramide generation and *CERS1* mRNA and protein were suppressed by FLT3-ITD [162]. Pharmacologic inhibition of FLT3 by crenolanib and sorafenib, rescued ceramide generation, increased the levels of *CERS1* mRNA and protein, and led to mitophagy and cell death in AML cells [162]. These effects substantiate ceramide metabolism as a potential novel target in *FLT3-ITD* AML [162]. Interestingly, co-treatment of ceramide and tamoxifen induced mitophagy in *FLT3-ITD* AML cells leading to a reduction in AML cell viability [168].

Recently, bioinformatic analysis of RNA-seq data of AML patients revealed that *FLT3-ITD* mutation is correlated with high *SPNS3* expression and relapse in AML patients [169]. The Spinster family is composed by SPNS1, SPNS2, and SPNS3. While SPNS1 is known to contribute to autophagy [170] and SPNS2 transports S1P [171] and has been recently implicated to promote tumor metastasis [172], not much is known about the exact function(s) of SPNS3 [173]. However, some insight into the role of SPNS3 in AML has emerged. For example, in an analysis of TCGA data from 155 AML patients, high expression of *SPNS2* and *SPNS3* in these samples correlated with poorer prognosis [174]. It was also found that *SPNS3* knockdown in MOLM-13 cells induced apoptosis and inhibited anti-apoptotic BCL2 and Mcl-1 [169]. 

Of additional relevance is the sphingolipid-modulating effects of resveratrol in the context of *FLT3-ITD* AML. Resveratrol is one of the most studied polyphenols derived from plants. It modulates the SL pathway in a variety of cancers, including hepatocellular carcinoma, gastric cancer, lung adenocarcinoma, and leukemia [175,176,177,178]. Resveratrol was previously shown to cause apoptosis in HL-60 cells due to the accumulation of ceramides and a reduction in *SPHK1* and *GCS* expression [178]. Building on this, a recent study assessed the independent and combinatorial effects of SKI-II (SPHK1 inhibitor) and PDMP (GCS inhibitor) with resveratrol in MOLM-13 and MV4-11 leukemic cells [179]. In line with previous observations, resveratrol decreased SPHK1 protein levels, as well as GCS. Inhibition of these enzymes with SKI-II and PDMP led to an additional decrease in cell viability, as well as increased apoptosis as measured by PARP and caspase-3 cleavage [179]. One interesting conundrum is the effect of resveratrol on SPT and the role played by SPT in the response to resveratrol treatment. In fact, while resveratrol elevated SPT levels [180], unexpectedly, the blockade of SPT with myriocin enhanced (rather than preventing) the anti-leukemic and proapoptotic effects of resveratrol. These findings are in line with previous evidence showing that the inhibition of SPT in AML with compound-2 exerts anti-leukemic effects [181] and support a pro-survival activity by SPT in AML and in response to resveratrol.

IV. S1P Receptors

Recent evidence also points to the involvement of S1P receptors in AML. S1PR_3_ specifically has been implicated in AML; however, its exact function is still controversial. In one report, it was found that *S1PR*_3_ overexpression in HSCs induced leukemia, pointing to high *S1PR*_3_ as an AML-inducing factor and a potential target for therapy [182]. Furthermore, *S1PR*_3_ gene expression was elevated in AML patient samples compared to subjects without AML further pointing to a role for S1PR_3_ as a potential leukemogenic factor [182]. However, this finding seems to oppose other studies which came to different conclusions [129,183,184]. First, *S1PR*_3_ expression was shown to be very low in normal hematopoietic stem and progenitor cells (HSPCs) and to be highest in mature myeloid cells, hinting to a potential role for S1PR_3_ in assisting myeloid differentiation. Indeed, overexpression of *S1PR*_3_ in HSCs stimulated myeloid differentiation in vitro and in vivo at the expense of the erythroid lineages via its activation of an inflammatory response. In line with these observations, a high expression of *S1PR*_3_ in leukemic cells derived from patients with AML was associated with a mature myeloid signature accompanied by a transcriptional inflammatory state [183]. The subset of leukemic stem cells with high *S1PR*_3_ were less functional due to S1PR_3_′s constraint on the self-renewal capacity. Moreover, S1PR_3_ activation in primitive AML cell lines led to differentiation of leukemic stem cells and their subsequent elimination [184]. Importantly, treatment of patient-derived xenografts (PDX) with the S1P receptor modulator FTY720 (fingolimod) [185] led to a significant reduction in the leukemic burden for 3 out of 12 PDXs and it reduced the leukemic stem cell frequency in a serial repopulation assay for 5 out of 7 samples tested. Since the mode of action of FTY720 involves both agonist and antagonist effects on S1PRs, the precise mechanism by which FTY720 curbed leukemic stem cells can not be extrapolated, but given its approved clinical use for multiple sclerosis, a possible repurposing for AML could be considered [183]. Indeed, FTY720 has also been shown to promote apoptosis of M2-AML cells by increasing levels of pro-apoptotic ceramide [186]. Altogether, these results point to a more complex role for S1PR_3_ than first inferred, specifically in the fraction of primitive AML cells compared to the entirety of the leukemic cell population. 

Of note and surprisingly, sphingosine, sphinganine, and ceramide levels were found to be higher in AML samples, with a ceramide/S1P ratio significantly higher in AML patient samples compared to control samples [187]. Similar changes in sphingosine, sphinganine, and ceramide levels were also found in blood samples from patients with multiple myeloma [188].

V. Additional Preliminary Links Between SLs and AML

The role and function of neutral sphingomyelinase 2 (nSMase2) has also been investigated in AML. Mutations in the *SMPD3* gene (encoding for nSMase2) have been reported in certain subsets of AML and ALL patient samples [189]. Interestingly, it was also found that reconstituting *SMPD3* in mouse tumor cells that did not express this gene led to increased cell death, suggesting that normal *SMPD3* counteracts pro-proliferative phenotypes associated with AML cells [189]. As nSMase is responsible for the conversion of SM to ceramide, changes in ceramide and/or SM could be of relevance. However, the biology and bioactive lipid mediator(s) downstream of *SMPD3* genetic alterations in AML have not yet been established. 

Other preliminary functional links between SLs and AML include a purported bioactive role for C1P via a yet unidentified C1P receptor in AML cells. First, AML cell lines KG1a, Hel, HL-60, U937, DAMI, and Jurkat were found to show enhanced migration when stimulated with 10μM or 20μM extracellular C1P [190]. Second, C1P (along with S1P) has been implicated as a chemoattractant for HSCs, as shown post-conditioning for bone marrow transplantation in lethally irradiated mice [191]. Irradiation leads to enrichment in proteolytic enzymes in the bone marrow, which in turn impairs the chemoattractant α-chemokine stromal-derived factor-1; therefore, it is hypothesized that there must be other factors that serve as chemoattractants which are not affected by proteolytic enzymes. The finding that C1P is a chemoattractant in this model and that it is resistant to proteolytic enzymes is thus relevant in this scenario [191]. Additionally, evidence points to the release of C1P by damaged cells after conditioning for bone marrow transplantation [192] and phosphorylation of an intracellular mitogen-activated protein kinase (MAPK) observed upon C1P stimulation in KG1a, Hel, HL-60, U937, DAMI, and Jurkat AML cell lines [190]. Therefore, these observations altogether hint at the presence of yet unidentified receptor(s) for C1P in these cells.

Finally and as briefly mentioned earlier, SPT has also been recently implicated in AML, and SPT inhibitors have been proposed as potential therapeutics [181]. In fact, inhibition of SPT by compound-2 (a novel SPT inhibitor [181,193]) not only caused the expected reduction in ceramide and SM, but also exhibited anti-tumor activity in a mouse model of AML. Similar effects were also observed with other novel SPT inhibitors [193].

VI. Therapeutic Strategies

**Figure 2:**
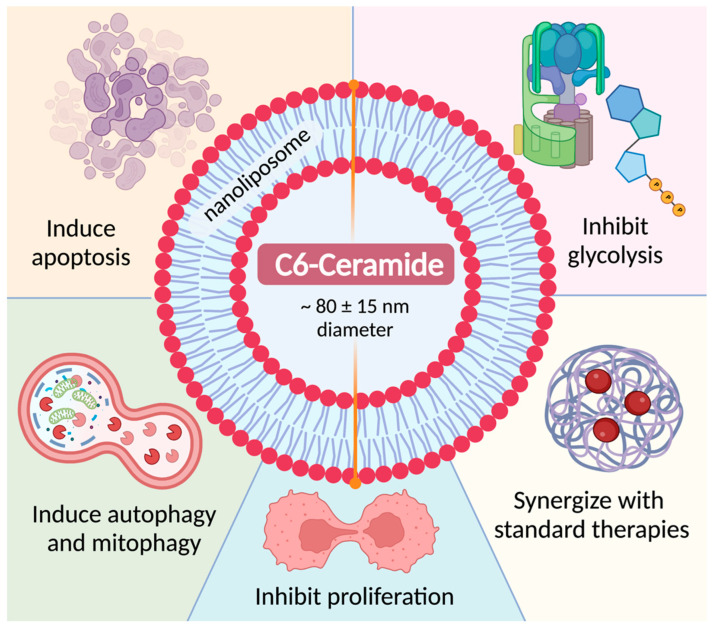
A summary of the effects of nanoliposomal C6-ceramide in hematological malignancies. Recent advances in nanoliposomal C6-ceramide therapy show that nanoliposomal C6-ceramide induces apoptosis, inhibits glycolysis, induces autophagy and mitophagy, inhibits proliferation, and synergizes with standard therapies across different hematological malignancies. Figure was created using BioRender.com (accessed on 18 October 2022).

One of the most advanced therapeutic strategies that capitalizes on the anti-tumor effects of ceramides, is the treatment with a nanoliposomal preparation of the short fatty acid chain and cell-permeable analog of ceramide, C6-ceramide [194,195]. Ceramide nanoliposomes (Ceraxa by Keystone Nano) completed a Phase I clinical trial in 2017 for advanced solid tumors and a clinical trial was also reportedly ready to open for patients with relapsed/refractory AML (ClinicalTrials.gov Identifier: NCT04716452). Nanoliposomal C6-ceramide has been found to exert anti-tumor effects in vitro and in vivo against a large number of cancers including leukemia and myeloma. In hematological malignancies, nanoliposomal C6-ceramide has been shown to exert anti-leukemic effects by inducing apoptosis, inhibiting glycolysis, inducing autophagy and mitophagy, inhibiting proliferation, and synergizing with current chemotherapies, which will be expanded upon as relevant in this review (Figure 2) [196,197,198,199].

Moreover, the combination of nanoliposomal C6-ceramide with the ceramide-inducing chemodrug vinblastine caused an escalated accumulation of ceramide and stronger apoptotic response in AML patient samples, due to the hydrolysis of C6-ceramide into Sph and in part to Sph reacylation to form endogenous ceramide [198]. Based on the effects in other cancers, studies have also proposed the use of nanoliposomal C6-ceramide as a powerful adjuvant therapy in combination with vinblastine, a microtubule inhibitor and blocker of the maturation stages of the autophagic process [200]. Considering its effects on autophagy and apoptosis, when tested in an in vivo model of *FLT3-ITD* AML, the combination of nanoliposomal C6-ceramide with vinblastine had synergistic effects, supporting other recent studies [198,201]. Additionally, nanoliposomal C6-ceramide was tested in combination with the flavonoids quercitin and 7,8-benzoflavone (BF). Quercitin is a polyphenol derived from anti-inflammatory blueberry extracts and led to a significantly greater decrease in viability of AML cell lines KG-1 and 32D-FLT3-ITD than either treatment alone [202]. BF was identified from a library of natural products as a compound that exacerbates the cytotoxic effect of nanoliposomal ceramide in AML cells [203]. Interestingly, the in vitro and in vivo combinatorial effect of BF with nanoliposomal C6-ceramide was observed in 32D-FLT3-ITD, but not C1498 cells, suggesting a cell-specific environment conducive to BF effectiveness. The authors suggested that the oxidative status of the cells or the levels of P-gp (and their effect on glycoSL synthesis) could potentially determine the effectiveness of BF in combination with ceramide nanoliposomes. 

Another SL modulator with therapeutic potential in AML is fenretinide (or 4-HPR). Fenretinide is a synthetic retinoid derivative and a known inhibitor of dihydroceramide desaturase [204,205,206,207]. Recently, fenretinide treatment of AML cell lines has been shown to downregulate anti-apoptotic BCL2, suppress tumor necrosis factor-α, as well as inhibit NF-κB [208]. In regard to SLs, fenretinide has also been shown to promote a significant increase in ceramide from sphingomyelin via activation of SMase [209]. Thus, fenretinide has the potential of increasing dihydroceramides through its inhibition of DES1 and/or ceramides through its activation of SMase. Fenretinide treatment led to cytotoxicity in KG-1, and parent and multi-drug-resistant (MDR) HL-60 AML cells, and this effect correlated with the accumulation of ceramide as well as a marked increase in reactive oxygen species [209]. This effect was interestingly not seen in K562 chronic myelogenous leukemia (CML) cells, which contrary to AML cells, did not produce ceramide in response to fenretinide treatment [209].

Also in the context of chemoresistance, the combination of SL modulating agents with current first-line therapies, such as the BCL2 inhibitor venetoclax, holds significant therapeutic value. While venetoclax is among the newer drugs approved for AML treatment in combination with low-dose cytarabine or hypomethylating agents [150,151], a significant obstacle to a long-term response to this therapy is resistance [210]. In addition to the sensitizing effect of SPHK1 inhibitor MP-A08 in combination with venetoclax discussed earlier [142], a new study also evaluated the effect of C6-ceramide nanoliposomes in combination with the venetoclax/cytarabine regimen in vitro in AML cell lines, ex vivo in AML patients samples, and in vivo in immunodeficient NRG and immunocompetent syngeneic mouse models [211]. In all experimental models, the combination therapy of C6-ceramide nanoliposomes with venetoclax/cytarabine exacerbated the antileukemic effects. In vitro, these were accompanied by enhanced apoptosis and autophagy. The triple combination also suppressed Mcl-1, an indicator of venetoclax resistance, and checkpoint kinase 1, associated with cytarabine resistance. The results, therefore, reveal a benefit brought in by the addition of C6-ceramide nanoliposomes to a standard of care, even in case of drug resistance. As mentioned earlier, a clinical trial looking at the addition of C6-ceramide nanoliposomes to the standard of care in patients with relapsed/refractory AML has been registered (ClinicalTrials.gov Identifier: NCT04716452).

#### 2.1.2. Acute Lymphocytic Leukemia (ALL)

Acute lymphocytic leukemia (ALL) is a hematological malignancy arising from the abnormal proliferation of lymphoid progenitor cells in the bone marrow, peripheral blood, and extramedullary organs [212]. ALL carrying the Philadelphia chromosome (Ph^+^) is the most common cytogenetically defined subtype of adult ALL, comprising 20-30% of all cases [213] but reaching 50% incidence in ALL patients older than 60 years of age [214]. The Ph^+^ subtype is characterized by a specific genetic footprint: the reciprocal translocation between the breakpoint cluster region (BCR) on chromosome 22 and the Abelson kinase gene (ABL1) on chromosome 9. The translocation gives rise to a shorter chromosome 22 named the Philadelphia chromosome (Ph) after the name of the city in which it was first discovered [215,216,217,218]. The fusion gene resulting from the translocation on the Ph chromosome encodes for the constitutively active tyrosine kinase BCR-ABL1 [219]. Depending on the location of the break within the BCR gene, fusion proteins of different sizes are formed such as BCR-ABL1 p210 (present in CML), p185 (present in ALL), or p190 (present in B-cell ALL) [220,221]. While the presence of *BCR-ABL1* in hemopoietic stem and progenitor cells is a hallmark of CML and it is sufficient to cause the development of CML [222,223,224], its presence in ALL confers an independent risk factor for a poor prognosis [225,226]. The discovery of the causal link between BCR-ABL and leukemia and the characterization of the tyrosine kinase activity linked to the fusion protein led to the development of the first successful targeted therapy with specific tyrosine kinase inhibitors (TKIs), such as imatinib [227]. Currently, first-line therapy for the Ph^+^ subtype of ALL is second-generation TKI, dasatinib along with chemotherapy [228]. Allogenic hematopoietic stem-cell transplantation is also often used alongside dasatinb treatment as a strategy to combat Ph+ ALL [229]. 

Evidence supporting the involvement of SLs in the response of Ph^+^ ALL cells to TKIs, such as imatinib, is only recently being reported in the literature. In particular, this points to the importance of SPHK1 and SPHK2 as downstream mediators of the oncogenic function of BCR-ABLl and as mediators of the cellular response to imatinib. To examine the role of SPHK1 in Ph^+^ ALL, B-cell progenitors isolated from either wild-type (WT) or *SPHK1*-knockout mice were transduced with the p185 form of the BCR-ABL oncogene, and then injected into C57BL/6 WT mice [230]. Absence of *SPHK1* caused a delay in the occurrence of leukemia in these mice [230]. In fact, among 29 mice receiving transduced cells isolated from *SPHK1* WT animals, 22 developed ALL and had a median survival of 42 days whereas, among 30 mice receiving cells isolated from *SPHK1*-knockouts, only 14 developed ALL and had a median survival of 100 days. Similar results were also obtained after injection of BCR-ABL-transformed *SPHK2* knockout cells in mice [231]. This suggests that SPHK1 and 2 play an important role in the development of Ph^+^ ALL. Furthermore, blockage of SPHK1 and/or SPHK2 using different small molecule inhibitors, such as SKI-I, SKI-II, and ABC294640 resulted in synergistic cell death of Ph^+^ ALL cell lines when combined with imatinib. These results highlight SPHKs as possible targets to optimize Ph^+^ ALL treatment [230,231]. Additionally, inhibition of SPHK2 on its own exerted toxic effects in Ph^+^ ALL cells both in cell culture and in a xenograft model [231]. These effects were possibly connected to the reduced levels of c-MYC triggered by inhibition of SPHK2. The link between SPHK2 and c-MYC was further reinforced since *SPHK2* deletion reduced the incidence of Ph^+^ ALL and c-MYC expression [231]. Finally, it was also reported that *SPHK1* gene expression was significantly higher in Ph^+^ ALLs compared to all other ALL samples [230] and that ALL patient samples have elevated SPHK2 protein levels compared to progenitor B-cells [231]. SPHK2 has also been implicated in the development of BCR-ABL independent ALL [232]. In a mouse study, *Sphk* 2^−/−^ mice showed significantly reduced rates of ALL development compared to wild-type mice, and in those *Sphk* 2^−/−^ mice that did develop ALL, fewer died from ALL compared to wild-type mice [232]. 

Additionally, a role for ceramide synthase-6 (CERS6) was recently uncovered in ALL, with CERS6 conferring resistance to BCL2 inhibitor ABT-737 [233]. In drug-resistant ALL cell lines, CCRF-CEM and MOLT-4, knockdown of *CERS6* increased cytotoxicity to ABT-737. In the same study, it was also shown that CERS6 binds directly to Fas death receptor, thus impairing the formation of death-inducing-signaling-complex (DISC), which then inhibits apoptosis. Additionally, C16:0-Ceramide levels were found to be higher in ALL cell lines, as a result of CERS6 elevation. Thus, CERS6 may serve as a biomarker for resistance in ALL [233].

Older evidence also implicates SLs in BCR-ABL independent ALL via the use of the DES1 inhibitor, N-(4-hydroxyphenyl)retinamide (fenretinide, or 4-HPR). Treatment with fenretinide was cytotoxic in ALL cell lines MOLT-3, MOLT-4, CEM, NALM-6, SMS-SB, and NALL-1 [234]. This finding provided further support for investigating the use of fenretinide in ALL, and since then, a few studies have reported on this drug particularly in pediatric ALL [235,236,237], with a clinical trial commenced in April 2010 using fenretinide for treatment of children with recurrent ALL (but also AML and non-Hodgkin’s lymphoma; NCT01187810—terminated due to supply issues). In connection with fenretinide’s function as a DES1 inhibitor, the cytotoxic function of dihydroceramide (supposedly accumulating when DES1 is inhibited) has also been directly explored. Induction of de novo dihydroceramide synthesis via treatment with dihydrosphingosine, fatty acids, or GT-11 (another DES1 inhibitor) induced accumulation of C22:0- and C24:0-dihydroceramide, associated with cytotoxicity in MOLT-4, CCRF-CEM, COG-LL-317h, and COG-LL-332h ALL cell lines [238]. This finding points to dihydroceramide as a contributing factor in fenretinide’s cytotoxicity in ALL cells.

#### 2.1.3. Chronic Myelogenous Leukemia (CML)

As mentioned, CML is characterized by the presence of the *BCR-ABL1* oncogene (p210) in myeloid cells (as opposed to lymphoid cells in ALL) initiating the Chronic Phase of the disease. This phase is generally kept in check for years with the use of TKIs, such as imatinib [215,222,223,224]. These drugs have been used successfully as first-line therapy for CML [239], but given the long-term duration of the treatment, instances of resistance are still common [240]. In order to overcome resistance to first-generation TKIs (like imatinib), second- and third-generation TKIs have been developed [241]. Currently, imatinib (first-generation TKI), dasatinib or nilotinib (second-generation TKIs), or bosutinib (third-generation TKI) are used for first line treatment of CML [242,243]. Studies linking SLs with TKIs in CML have both invoked a SL response in mediating the cytotoxic effects of TKIs as well as pointed to changes in SL enzymes in contributing to the insurgence of TKI resistance. 

SL metabolism was shown to be involved in the cytotoxic effects of imatinib in different CML cell lines. In fact, ceramide accumulation was reported following treatment of the CML cell line K562 with cytotoxic doses of imatinib [244]. Importantly, preventing ceramide accumulation by downregulating the enzyme responsible for imatinib-induced ceramide formation (in this case CERS1) prevented in part the cytotoxic effect of imatinib. Similar observations were also reported in LAMA84 cells [245]. A more recent study also revealed that imatinib activates the SL pathway not only at the level of CERS1, but also by activation of SPT, the enzyme responsible for initiating SL biosynthesis [246]. Interestingly, the SPTLC1 subunit of the SPT complex was found to be phosphorylated at Tyr^164^ by ABL leading to inhibition of SPT activity. Treatment of *BCR-ABL1* positive cell lines K562 and LAMA-84 with the BCR-ABL inhibitor imatinib led to SPTLC1 tyrosine dephosphorylation which in turn increased SPT activity [246]. This supports a role for ceramide accumulation via the de novo pathway upon imatinib treatment [246]. Additionally, in K562 and LAMA-84 cells, an inversed correlation between the myeloid differentiation factor, interferon regulatory factor-8 (IRF8), and acid ceramidase (AC) was reported [247]. While it was shown that normally IRF8 binds to *ASAH1*’s promoter and represses *ASAH1* transcription, in CML cells, IRF8 levels are markedly low allowing for *ASAH1* expression. When expression of *IRF8* was restored, AC levels dropped with a concomitant accumulation of C16:0-ceramide and sensitization of CML cells to Fas ligand-induced apoptosis in vitro and CML development in vivo [248]. Importantly, modulation of AC, independently of IRF8, was able to significantly affect sensitivity to Fas ligand, highlighting the functional importance of AC.

Complementary observations revealed that altered SL metabolism is also involved in the mechanism of resistance to imatinib. In fact, it was shown that, in imatinib-resistant K562 and LAMA84 cells, resistance to the drug was connected to failure of the cells to accumulate ceramide in response to imatinib [244,249]. In these cells, lack of ceramide accumulation was due to the prompt conversion of this cytotoxic SL into pro-survival S1P through the enhanced expression of *SPHK1*. Importantly, partial inhibition of *SPHK1*/SPHK1 with siRNA or pharmacological agents sensitized-resistant cells to the cytotoxic effects of imatinib, while *SPHK1* overexpression in sensitive cells prevented imatinib-induced apoptosis. 

The mechanism by which SPHK1/S1P induces resistance to imatinib might involve the upregulation of BCR-ABL1 protein levels, independently of *BCR-ABL1* mutations. In fact, a study showed that the protein stability of BCR-ABL1 was increased in imatinib-resistant K562 and LAMA84 cells compared to the sensitive counterparts [250]. This phenotype was reverted by the downregulation of *SPHK1* and reduction of S1P in resistant cells. In particular, reducing S1P levels led to activation of the protein phosphatase 2A and the subsequent dephosphorylation and proteasomal degradation of BCR-ABL1. Of interest, another study revealed that BCR-ABL1 itself drives the upregulation of *SPHK1* in imatinib-resistant cells [251], thus pointing to the existence of a positive feedback loop between BCR-ABL1 and SPHK1 whereby BCR-ABL1 promotes *SPHK1* expression and SPHK1 in turn increases BCR-ABL1 by allowing its stabilization.

Imatinib resistance has been also associated with upregulation of GCS, the enzyme that converts ceramide into glucosylceramide by adding a molecule of glucose [249]. Indeed, expression of *UGCG* (gene name of GCS) was shown to be increased in imatinib-resistant K562 cells and pharmacological inhibition of the enzyme with PDMP sensitized these cells to the drug. Elevated expression of *SPHK1* and *UGCG* has been linked also to resistance to nilotinib, and similar to imatinib-resistant cells, inhibition of these enzymes sensitized nilotinib-resistant K562 cells to the drug [252]. In addition, it was also reported that multi-drug resistance is associated with higher *UGCG* expression in K562-derived cell lines, and inhibition of GCS with d-threo-EtDO-P4 decreased the expression of P-gp (MDR1/ABCB1) and sensitized these resistant cells to chemotherapy-induced apoptosis [253]. Altogether, these observations support the concept that targeting different steps of the SL pathway (in particular SPHK1 and GCS) may be a beneficial strategy to overcome TKI resistance in CML.

Analysis of SL levels in CD34^+^ cells derived from the bone marrow of patients who have advanced to the aggressive and refractory blast-phase of CML showed that ceramide levels in leukemic CD34^+^ cells were significantly lower than in normal CD34^+^ cells. The extent of ceramide reduction in blast-phase CML cells correlated with the level of resistance to TKIs [254]. Apoptosis of multiple CML cell lines including blast-phase CML CD34^+^ cells was achieved by increasing ceramide level with the addition of the short chain ceramide analog C2-ceramide or pharmacological inhibition of ceramide metabolizing enzymes with PDMP and SKI-II [197]. Synergistic effects against blast-phase CML progenitor cells were observed with a combination of dasatinib or nilotinib with either C2-ceramide or PDMP and SKI-II. Thus, the elevation of ceramide induced apoptosis and rescued sensitivity to TKIs [254]. The sphingolipidomics profile was also recently analyzed in plasma from patients at different stages of CML, including TKI-resistant patients, and it was found that ceramide levels were increased in TKI-resistant patients while SM and S1P levels were decreased in the chronic- and accelerated-phase CML [255]. These results seem to be discordant from sphingolipid levels measured in primary cells, particularly in the case of TKI resistance and this might be due to measurements in plasma versus cells [254]. 

In another recent study, the interaction between SPHK1/S1P and sirtuin 1 (SIRT1) was investigated in CML cells, whereby SPHK1/S1P induced *SIRT1* expression [256]. *SIRT1*, a nicotine adenine dinucleotide-dependent protein deacetylase, is an important regulator of cellular metabolism and bioenergetics, and it is constitutively expressed in CML [257]. SIRT1 inhibition sensitizes CML leukemic stem cells to TKI treatment through p53 activation [258]. Addition of either SKI-II, a specific inhibitor of SPHK1, or EX527, a specific inhibitor of SIRT1, blocked growth of K562 cells. The combination of both inhibitors had a synergistic effect in blocking growth and survival of CML cells [259]. While the effect of the combination of SKI-II and EX527 was not determined in the presence of TKI, it is noteworthy that this combination induced apoptosis of cells with the T-315I mutation, a major contributor to CML and Ph^+^ ALL resistance to TKIs. Altogether, these observations therefore suggest the possible benefit of targeting the SPHK1/S1P/SIRT1 axis as a novel anti-leukemic treatment strategy particularly in case of resistance to TKIs [259]. 

#### 2.1.4. Chronic Lymphocytic Leukemia (CLL)

CLL is characterized by an abundance of lymphocytes in the blood, specifically CD5^+^ B-cells [260]. It is the most common type of leukemia in western countries (and comparatively less common in eastern countries), and 80% of patients present with genetic aberrations, such as mutations in *TP53, NOTCH1*, or *SF3B1* [260,261,262]. Some of the current treatment options for CLL consist of BCL2 inhibitor Venetoclax, Bruton tyrosine kinase inhibitor ibrutinib, and the standard combination of fludarabine, cyclophosphamide, and rituximab [263].

SL metabolism has previously been shown to support survival of CLL cells, partly in response to known CLL survival stimuli. Engagement of B-cell receptor, interleukin-4, and CD40 ligand are all different stimuli promoting the survival of CLL cells [264,265,266,267,268,269,270]. When primary CLL cells were stimulated by engagement of the B-cell receptor, there was a decrease in ceramide levels and increase of glucosylceramide formation [264]. Upon B-cell receptor stimulation, levels of GCS were significantly increased. Treatment with inhibitors of B-cell receptor pathway, CAL-101 and PCI-32765, significantly lowered *UGCG* expression and sensitized CLL cells to treatment with BCL2 inhibitor ABT-737, increased levels of ceramide and induced apoptosis, further supporting a role for SLs in survival of these cells [264]. 

Additionally, modulation of S1PR_1_ seems to play a role in sequestering CLL cells in protective niches and extending their survival [271]. Normally, engagement of S1PR_1_ by S1P guides the egress of lymphocytes from lymph nodes into circulation [271,272,273,274,275]. However, signaling via B-cell receptor and CD40 in CLL cells has been shown to inhibit S1PR_1_ preventing cells from getting into circulation where they would be ultimately more vulnerable to cytotoxic drugs [276]. 

In the last five years, roles for SLs in anti-tumor activity by Natural Killer T (NKT) cells have also been investigated. α -galactosylceramide is presented to NKT cells by CD1d on B-cells, promoting their elimination by NKT cells [277]. Importantly, CD1d expression in B cells from CLL patients is decreased, as well as the frequency of NKT cells, with detrimental consequences [277]. Therefore, strategies to enhance NKT cell activity would be beneficial to CLL patients. Importantly, α-galactosylceramide was shown to enhance NKT-cell-mediated apoptosis of CLL cells [277,278,279]. Additionally, retinoic acid and the retinoic acid receptor-α agonist AM-580 was found to induce CD1d expression ex vivo in B-cell CLL cells from patients, promoting activation of cytotoxic invariant natural killer T (*i*NKT) cells [278]. 

Treatment with nanoliposomal C6-ceramide has also been a valuable approach in CLL, as it has been in other hematological malignancies. Nanoliposomal C6-ceramide treatment was found to inhibit *GAPDH* message and protein in CLL cell line JVM3, in primary cells from CLL patients, as well as in a mouse model of CLL [196]. Inhibition of GAPDH reduced glycolysis, which is highly utilized by cancer cells [280]. In addition to GAPDH, nanoliposomal C6-ceramide treatment also reduced the levels of phosphorylated STAT3 in PBMCs derived from CLL patients [281]. Phosphorylated STAT3 is critical for survival of CLL cells by promoting transcription of STAT3 regulated genes such as anti-apoptotic BCL2, BCL-x_L_, and more [282]. STAT3 inhibitors have been explored for treatment of CLL in the past and have been found to reduce drug resistance [283]. Thus, the effect of nanoliposomal C6-ceramide on phosphorylated STAT3 provides an important proof-of-principle in supporting the effectiveness of this treatment strategy for CLL. 

Another mean that has been investigated to promote apoptosis of CLL cells is to induce lysosome membrane permeabilization (LMP). One mechanism proposed to explain the cytotoxic effect of LMP in cancer cells, including CLL cells, is the inhibition of lysosomal acid sphingomyelinase (aSMase) [284,285,286]. Siramesine, a lysosomotropic agent, blocks the binding of aSMase with its cofactor bis(monoacylglycero)phosphate in the lysosome, which leads to their destabilization [286]. Other reports have also implicated endogenous levels of Sph priming CLL cells to LMP. In fact, it has been shown that CLL cells have increased endogenous Sph levels and exogenous addition of this SL (on top of the already increased levels) induced LMP in primary CLL cells, but not in healthy B-cells [287]. Thus, it is possible that the increased Sph levels in CLL cells uniquely sensitize them to LMP, causing apoptosis of CLL cells [287]. 

Mechanisms of resistance to apoptosis linked to SLs have also been explored in PBMCs isolated from CLL patients, and it was found that resistance to standard therapies (such as fludarabine, rituximab, and cyclophosphamide) was associated with an upregulation of GCS and decreased levels of ceramide [288]. Inhibition of GCS with PDMP ameliorated resistance to fludarabine [288]. While promising, these results need to be confirmed using more specific inhibitors (such as eliglustat) or with genetic manipulations (siRNA/CRISPR). 

#### 2.1.5. Large Granular Lymphocyte (LGL) Leukemia

LGL leukemia is a rare malignancy in which there is an expansion of NK cells (NK-LGL) and/or T-cells (T-LGL) [289]. Currently, survival is at a median of 9 years and there is a need for novel effective therapies [290,291]. STAT3 mutations are present in up to 40% of cases of T-LGL leukemia [292]. The current treatment regimen for LGL leukemia consists mostly of immunosuppressive agents such as methotrexate, cyclosporine A, and/or cyclophosphamide [293]. 

Several studies have linked S1P signaling to LGL leukemia [294,295,296]. S1P levels are high in the peripheral blood of NK-LGL leukemia patients, and this has been attributed to overexpression of *SPHK1* in PBMCs of these patients [295]. In one study, inhibition of SPHK1 with SKI-178 resulted in increased ceramide levels and decreased S1P levels, ultimately leading to apoptosis [295]. This was accompanied by halted JAK/STAT signaling [295]. Constitutive activation of JAK/STAT and mutant STAT3 are signatures of LGL leukemia and are involved in the progression of the disease [297]. Therefore, the link between SPHK1/S1P and JAK/STAT signaling is of particular relevance. 

AC was also shown to be overexpressed in T-LGL leukemic cells from patients, and its inhibition by N-oleoylethanolamine led to increased apoptosis compared to PBMCs from healthy samples [294]. Hence it is possible that the higher expression of both AC and SPHK1 in LGL leukemia cells is responsible for the larger production of S1P which in turn provides a proliferative/survival advantage. 

In addition to SPHK1, SPHK2 has also been implicated in survival of both NK- and T-LGL leukemic cells. SPHK2 inhibitors ABC294640 and K145 downregulated Mcl-1 and prompted apoptosis in both cell lines and patient samples [298]. 

Expression of S1PRs is altered in LGL leukemic cells, with S1PR_1_ being downregulated and S1PR_2_ and S1PR_3_ not expressed; on the other hand, S1PR_5_ was found to be overexpressed in T-LGL leukemic cells as compared to normal CD8^+^ cells [294]. Whether or not increased expression of S1PR_5_ has a functional role it is not known, however treatment with FTY720 of freshly isolated PBMCs from T-LGL patients induced apoptosis [294]. Given the ambivalent effect of FTY720 as both agonist and antagonist of S1PRs, and considering that FTY720 has been also shown to activate S1PR_5_ [299], a definitive conclusion on the functional contribution of S1PR_5_ cannot be drawn at this time. 

In addition to increased S1P, changes in select SM species were also uncovered by a large sphingolipidomic study that measured 33 SLs in sera from 50 patients. C20:0-SM, C22:0-SM, and C24:0-SM were found to be significantly less abundant in LGL leukemia samples compared to samples from normal subjects [296]. The authors proposed that this signature may serve as a biomarker and diagnostic tool in identifying LGL leukemia in patients. Additionally, C24:0-SM and C26:1-SM were positively associated with levels of normal and mutant STAT3 [296]. This may indicate that while levels of C24:0-SM may be lower in LGL leukemia, this particular SM may still be associated with LGL leukemic survival through its correlation with mutant STAT3. Further studies would be required to conclusively address the contribution of C24:0-SM in LGL leukemia. 

S1P has also been found to have a linear relationship to normal, but not mutant STAT3 [296]. This is an interesting observation, considering that mutant STAT3 contributes to survival of T-LGL leukemic cells [300,301]. However, considering the high levels of S1P and STAT3 in these patients, perhaps high STAT3, even if not mutated, contributes to the pathogenesis of T-LGL leukemia. 

Much like other hematological malignancies, nanoliposomal C6-ceramide is a promising therapeutic option for LGL leukemia. In a rat model of NK-LGL leukemia, treatment with nanoliposomal C6-ceramide-induced apoptosis and decreased survivin [302]. Survivin is known to support progression of tumor development as well as to inhibit apoptosis [303] and it is highly expressed in the peripheral blood of NK-LGL leukemia patients [302]. Therefore, the effect of nanoliposomal C6-ceramide on survivin holds promise for nanoliposomal C6-ceramide as a future treatment strategy in LGL leukemia. 

#### 2.1.6. Adult T-Cell Leukemia (ATL)

ATL is a type of leukemia that is initiated by infection with human T-cell lymphotrophic virus-1 (HTLV-1), which is the first human retrovirus to be discovered [304,305]. Nearly 10 million people are infected with HTLV-1 each year, with infections endemic to Japan, the Caribbean, the Middle East, parts of Africa, and Central America [306]. ATL is an aggressive form of leukemia with a survival rate of less than a year [307]. The prognosis for ATL is complicated by high rates of chemotherapeutic resistance and infections [307]. Given the association with HTLV-1, current therapeutic regimens take advantage of anti-viral compounds, such as zidovudine and IFN-α, in addition to allogeneic HSC transplantation [307]. The standard combination of chemotherapeutics for aggressive ATL is vincristine, cyclophosphamide, doxorubicin, and prednisone [308]. 

While some data have shown that all-trans retinoic acid (ATRA) may be a favorable treatment option for ATL [309], the response to ATRA was partial and not as encouraging. ATRA is a retinoid with anti-cancer properties in a variety of cancers; however, resistance to ATRA is also a common response of cancer cells [310]. In case of resistance or suboptimal response to ATRA, synthetic retinoids are often considered, such as ST1926 [311] and fenretinide [312]. ATRA and synthetic retinoids have been shown to modulate SL metabolism in several different cancer cell types, with breast cancer cells and acute promyelocytic leukemias among the most studied [313,314,315]. Recently, treatment of ATL cells with ST1926 was shown to increase de novo ceramide synthesis [316] and, differently from fenretinide, it did not inhibit DES1. The stimulatory effect on de novo synthesis led to accumulation of pro-apoptotic ceramide, suggesting that restrained de novo ceramide synthesis may contribute to ATL cell survival and that remediating this with ST1926 could represent an effective treatment for ATL through its induction of apoptosis. On the other hand, another study seems to dispute the specificity of action of the other synthetic retinoid, fenretinide, against ATL. In fact, fenretinide causes an apoptotic response in both HTLV-1^+^ and HTLV-1^−^ cells. HTLV-1^−^ cells showed higher sensitivity to fenretinide and fenretinide caused accumulation of ceramide only in HTLV-1^−^ [312]. Hence the jury is still out on whether fenretinide can be used to specifically treat ATL, or whether more specific treatments or exploration of other synthetic retinoids could prove to be more beneficial in ATL. 

### 2.2. Lymphoma

Lymphoma is broadly categorized by abnormal proliferation of lymphoid precursors or mature lymphoid cells, and by tumors in lymph nodes, tonsils, salivary glands, or other associated regions in the head and neck [317]. They are classified into two distinct groups: Hodgkin lymphoma and non-Hodgkin lymphoma (NHL). Hodgkin lymphomas are characterized by the presence of large, multinucleated B-cell derived Reed–Sternberg cells, with activation of JAK/STAT signaling pathways as well as NF-κB [318]. If Reed–Sternberg cells are not present, then the lymphoma is classified as NHL. Diffuse large B-cell lymphoma (DLBCL) is the most common subtype of NHL, followed by follicular lymphoma [319]. Less commonly occurring NHLs include T-cell and primary effusion lymphomas [320,321]. The standard treatment strategy for Hodgkin lymphoma is the chemotherapeutic regimen of doxorubicin, bleomycin, vinblastine and dacarbazine [322]. NHL is currently treated with radiotherapy, chemotherapeutics, and immunotherapies, with treatment including rituximab with a regimen of cyclophosphamide, vincristine, doxorubicin or epirubicin, and prednisone [323,324]. 

SL metabolism is known to play a role in the cytotoxic response to therapeutics in lymphoma. Specifically, *RUNX* genes (*RUNX1-3*), which partially act as oncogenes but also have tumor-suppressing abilities depending on the context in which they are found [325], have been previously shown to regulate SL metabolism via transcriptional regulation of the SL genes *Sgpp1*, *Ugcg*, and *St3gal5* in murine NIH-3T3 fibroblasts [326]. Transcriptional downregulation of *Sgpp1* and upregulation of *Ugcg* by *Runx1* have also been established in murine lymphomic cells [327]. In addition to the reported targets, overexpression of *RUNX1* was also found to promote the release of S1P from T-cell lymphoma cell lines, promoting survival [327]. Upon treatment with dexamethasone, *RUNX1* overexpressing cells evaded apoptosis, thus indicating that RUNX1 opposed dexamethasone treatment and promoted drug resistance [327]. Overall, this posits transcriptional regulation of SL genes *SGPP1* and *UGCG* by *RUNX1* as a potential link to drug resistance and survival of lymphoma cells. 

The roles of S1PRs have also been investigated. Expression of S1PR_2_ seems to be associated with an antitumor effect. In fact, *S1PR*_2_ is found mutated and silenced in some cases of DLBCL [328]; the mutations were associated with pro-proliferative effects, while expression of WT-S1PR_2_ led to lymphomic cell death [329]. Similarly, *S1pr2*^−/−^ mice develop B-cell lymphoma, thus supporting that disruption of S1PR_2_ seems to have lymphoma-promoting effects [330]. Additionally, expression of specific *S1PRs* seems to be associated with particular locations of lymphomas [331]. In fact, patient samples of lymphomas from secondary lymphoid organs (SLOs) had higher expression of *S1PR*_2_, while samples from ocular adnexal lymphomas (OAL) had higher *S1PR*_3_ expression.

Recently, genome-wide microRNA expression profiling of peripheral T-cell lymphomas revealed that miRNA signatures that regulate SL signaling were altered specifically in angioimmunoblastic T-cell lymphomas, compared to other peripheral T-cell lymphomas, and implicated dysregulation of SL signaling in supporting the survival of angioimmunoblastic T-cell lymphoma [332]. One of the upregulated miRNAs was specifically associated with the repression of *S1PR*_1_, hinting to a role for low S1PR_1_ in lymphoma [332]. Since angioimmunoblastic T-cell lymphomas are aggressive, and the overall patient survival is only about 3 years [333], the possibility that SL dysregulation is relevant for the maintenance of these cells provides an interesting potential avenue for the development of novel specific therapeutics. 

Ceramide has also been implicated in the apoptosis and cytotoxicity of lymphoma cells. When lymphoma cells are deprived of IL-2 (a factor that promotes their survival [334]), ceramide accumulates following activation of aSMase prior to induction of apoptosis [335,336]. This seems to be in line with a previous study showing that rituximab, an anti-CD20 antibody used for the treatment of B-cell non-Hodgkin’s lymphoma [337,338], causes an increase in acid sphingomyelinase [339]. 

SL regulation and its therapeutic applications have been also explored in the context of primary effusion lymphoma (PEL). PEL is an aggressive variant of DLBCL, causally associated with Kaposi’s Sarcoma-associated herpesvirus (KSHV) [340,341,342]. In the PEL (and KSHV^+^) cell line, BCBL-1, targeting SPHK with ABC294640 (a SPHK inhibitor with more selectivity for SPHK2) or downregulation of *SPHK2* with siRNA led to ceramide accumulation, caspase activation, inactivation of survival pathways such as ERK, AKT and NF-KB, and apoptosis [343]. Interestingly, ABC294640 also increased the expression of lytic genes which may also contribute to its cytotoxic effect [343,344]. Importantly, administration of ABC294640 in vivo caused tumor regression of already established BCBL-1 xenografts, suggesting therapeutic potential [343]. Complementary to this, transcriptomic changes induced by exogenously applied dhC16:0-ceramide in BCBL-1 cells revealed elevated proinflammatory signatures and markedly upregulated tumor suppressor genes [345]. The discussed functional association between SPHK2 and the regulation of ceramide in PEL cell models encouraged further investigation into the therapeutic applications of ABC294640. Indeed, a clinical trial testing ABC294640 in DLBCL (NCT02229981) was initiated, but later withdrawn due to a lack of recruitment (Table 1).

Newly synthesized ceramide analogs comprised of sulfonamide or amide in the backbone or sidechains of ceramide were also recently explored in PEL [361]. The majority of these ceramide analogs exerted anti-lymphoma effects and led to apoptosis, cell cycle arrest, and inhibition of cellular proliferation of BCBL-1 cells [361]. Treatment of PEL cell lines with ceramide analogs caused downregulation of *CDCA3* (cell division cycle-associated 3) and *AURKA* (Aurora kinase) [361]. These results become relevant as *CDCA3* has been recently found to contribute to leukemogenesis [362] and Aurora Kinase was found to be increased in AML [363]. These genes were also essential for survival of PEL cells, and in addition to downregulation upon treatment with ceramide analogs, their expression was also found to be regulated by *SPHK2* whereby knockdown of *SPHK2* led to reduction of *AURKA* and *CDCA3* expression in PEL cells [361]. Therefore, one can expect that a two-hit treatment, with ceramide analogs and SPHK2 inhibitor, would exert the most robust effect. 

Modulation of SLs, particularly S1P by SPHK1, has been also linked to the alteration of the NKT immune response against mantle cell lymphoma (MCL) cells [364]. Both *SPHK1* and *SPHK2* were found to be elevated in the MCL cell line SP53, and inhibition of S1PR_1_ or knockdown of *SPHK1* in SP53 and Jeko-1 cells enhanced NKT cell activation and NKT response against MCL. Mechanistically, loss of SPHK1 increased cardiolipin; in fact, this lipid was shown to bind to antigen presenting CD1d and its exogenous addition enhanced activation of NKT cells [364]. As MCL is an aggressive subtype of NHL, enhancing NKT cell-mediated killing of MCL cells could represent a useful strategy to reduce tumor burden.

SLs also play a role in the transcriptional regulation of angiogenesis in lymphoma. Specifically, *SPHK1* and S1P were increased in primary samples of DLBCL [365]. Analysis of DLBCL datasets showed that *SPHK1* correlated with *VEGF*, a gene associated with angiogenesis, as well as with the genetic signature associated with tumor vasculature [365]. A similar analysis also revealed that *S1PR*_1_ expression was associated with genes that comprised the tumor vasculature signature [365]. Based on this, treatment with an antagonist of S1PR_1_, Siponomid, reduced angiogenesis and tumor growth in a DLBCL mouse model, indicating that S1P-S1PR_1_-mediated angiogenesis is a potential therapeutic target to subdue DLBCL [365]. 

### 2.3. Multiple Myeloma

Multiple myeloma is a cancer of plasma cells, which expand uncontrollably and produce abnormal monoclonal immunoglobulins [366]. Current treatments for multiple myeloma include a combination of dexamethasone with an immunomodulatory agent such as lenalidomide, and a proteasome inhibitor such as bortezomib [367]. Previous literature has supported a role for S1P in enhancing migration and adhesion of multiple myeloma cell lines [368], as well as promoting survival of these cells and resistance to dexamethasone treatment [369]. 

In addition to S1P, altered ceramide metabolism has also been linked to multiple myeloma with a main focus on aSMase; however, as discussed below, the understanding of the relationship between expression and activity of aSMase in multiple myeloma is still incomplete. ASMase was found to be upregulated in primary multiple myeloma cells isolated from plasma samples [370], as well as upon treatment with green tea extract in primary multiple myeloma patient cells, and myeloma cell lines [371]. Moreover, treatment with melphalan and bortezomib led to upregulation of aSMase in cells and in their exosomes [370]. This is interesting as multiple myeloma is known to be resistant to melphalan and bortezomib [372] and upregulation of aSMase could be a molecular response to induce such resistance. Indeed, exosomes that were high in aSMase were able to confer resistance to cells that were previously responsive to treatment [370]. However, while analysis of blood samples from multiple myeloma patients showed that ceramide, sphingosine, and sphinganine levels were significantly higher in these patients compared to healthy patients [188], aSMase activity was significantly lower [188]. ASMase activation has been also implicated in the induction of cell death by the major polyphenol in green tea extract, EGCG [(−)-epigallocatechin-3-O-gallate] [348,371]. In this study, activation of aSMase in response to the interaction between EGCG and the 67-kDa laminin receptors (67LR) altered lipid-raft clustering [371] and inhibited the prosurvival activity of tyrosine kinase receptors both in primary multiple myeloma cells and in the multiple myeloma cell line, U266 [348]. Additionally, *SPHK1* expression was higher in multiple myeloma cell lines U266, ARH-77, RPMI8226, as well as in primary patient cells [348]. Blockade of SPHK1 activity with the competitive inhibitor safingol acted in synergy with EGCG to induce cell death. This effect correlated with the reduced activation of tyrosine kinase receptors and activation of the death-associated protein kinase 1 (DAPK1) [348,371,373]. Hence, the combination of EGCG and safingol (or other SPHK1 inhibitors) could represent a novel promising strategy.

Similar to *SPHK1*, *SPHK2* was also found to be overexpressed in primary CD1381 multiple myeloma cells, as well as in 7 different myeloma cell lines [374]. *SPHK2* inhibition with short-hairpin RNA or ABC294640 produced pro-apoptotic effects [374]. Moreover, the combination of bortezomib, a proteasome inhibitor and FDA approved drug for multiple myeloma, with inhibition of SPHK2 synergistically increased ER stress and apoptosis [114]. However, despite the purported functional link between S1P/SPHK2 and survival of multiple myeloma cells, SL analysis of blood samples from 83 multiple myeloma patients at different stages of disease and 17 healthy patients found no difference in S1P levels in multiple myeloma patients compared to healthy patients [188].

C6-ceramide supplementation, similarly to other hematological malignancies, has been found to inhibit the proliferation of multiple myeloma cells, as well as to induce apoptosis [375]. In the multiple myeloma cell line, OPM2, C6-ceramide treatment led to an increase in PARP cleavage, as well as an increase in caspase 3/9, indicating the induction of caspase-mediated apoptosis [375]. Nanoliposomal C6-ceramide has not yet been investigated in multiple myeloma, but it is fair to expect that nanoliposomal C6-ceramide treatment would exert similar effects on proliferation and apoptosis as observed with C6-ceramide supplementation. Increased synthesis of C16:0-ceramide was also observed upon BCL-2/BCL-x_L_ inhibitor Navitoclax (ABT-263) treatment of myleoma cell line RPMI8226. The treatment activated CerS-mediated C16:0-ceramide production and promoted apoptosis [376].

## 3. Conclusions

**Figure 3:**
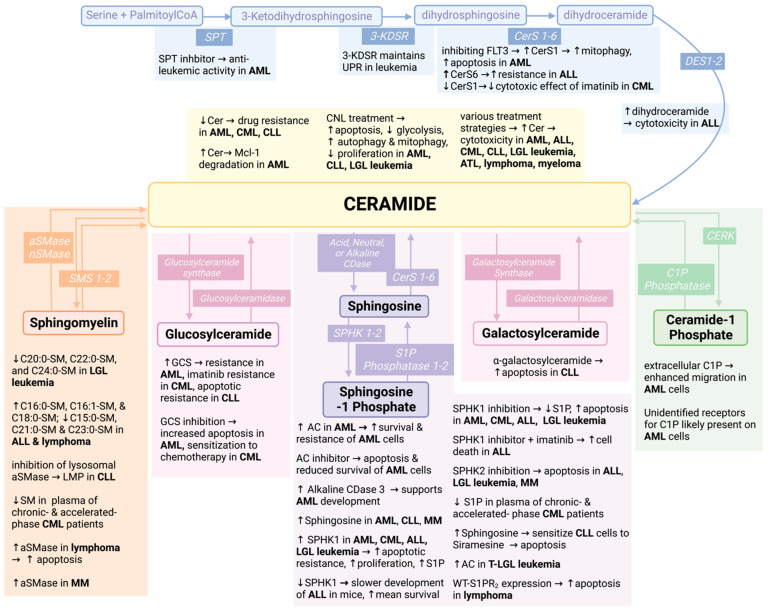
An overview of many recent advances on sphingolipid involvement in hematological malignancies. Up arrows indicate high/increased expression or levels; down arrows indicate low/decreased expression or levels; forward arrows indicate “associated with”. Figure created with BioRender.com (accessed on 21 October 2022). Sphingolipid pathway abbreviations—SPT: serine palmitoyltransferase; 3-KDSR: 3-ketodihydrosphingosine reductase; Cer: ceramide; CerS: ceramide synthase; DES: dihydroceramide delta(4)-desaturase; aSMase: acid sphingomyelinase; nSMase: neutral sphingomyelinase; SM: sphingomyelin; SMS: sphingomyelin synthase; AC: acid ceramidase; CDase: ceramidase; SPHK: sphingosine kinase; S1P: sphingosine-1-phosphate; S1PR: sphingosine-1-phosphate receptor; C1P: ceramide-1-phosphate; CERK: ceramide kinase; GCS: glucosylceramide synthase; CNL: C6-ceramide nanoliposome. Hematological malignancy abbreviations—AML: acute myeloid leukemia; ALL: acute lymphocytic leukemia; MM: multiple myeloma; CML: chronic myeloid leukemia; LGL: large granular lymphocyte; CLL: chronic lymphocytic leukemia; ATL: adult T-cell leukemia; LMP: lysosomal membrane permeabilization.

In the preceding sections, a multitude of roles for SLs and SL metabolizing enzymes have been discussed in the context of hematological malignancies, with many of those relationships summarized in Figure 3. There have been some overall trends identified, for example, the therapeutic value of nanoliposomal ceramide across different leukemias (the effects of which are summarized in Figure 2). There have also been recurring and multifaceted roles identified for acid ceramidase, acid sphingomyelinase, sphingosine kinase, glycosphingolipids, S1P, and more. The discovery and understanding of alterations within the sphingolipidome (expression and activities of SL enzymes as well as SL levels) in hematological malignancies have provided multiple novel therapeutic targets, especially in AML. Importantly, inhibitors of SL metabolism have played a significant role in deciphering functional connections between SLs and hematological malignancies, with some of these also investigated therapeutically (Table 1). The research in this field is rapidly advancing with ceramide-based clinical trials in the works, and the coming years will likely be crucial to assert the clinical benefits of SL-based therapeutics. 

## Figures and Tables

**Table 1:** Sphingolipid inhibitors used in the context of hematological malignancies referenced in this review.

Inhibitory Activity	Pharmacological Agent	Malignancy	Clinical Trial	Status
SPHK	SKI-I [346]	ALL [231]		
SPHK1	SKI-II [346]	ALL [230]		
	SKI-178 [141]	AML [141] LGL Leukemia [295]		
	MP-A08 [115]	AML [145]		
	Safingol [347]	Multiple Myeloma [348]		
	Dimethylsphingosine [349]	AML [138]		
SPHK2	ABC294640 [350]	Lymphoma [344]	NCT02229981	Terminated: lack of recruitment
		LGL Leukemia [298]		
		ALL [231]		
		Multiple Myeloma [351]	NCT02757326	Terminated: funding
	K145 [352]	LGL Leukemia [298]		
SPT	compound-2 [181]	AML [181]		
	Myriocin [353]	AML [180]		
GCS	PDMP [354]	AML [158,160]		
		CLL [288]		
		CML [249]		
	d-threo-EtDO-P4 [355]	CML [253]		
DES1	N-(4-hydroxyphenyl)retinamide (4-HPR, Fenretinide) [204]	AML [208]	NCT01187810 NCT00104923	Terminated: drug supply Completed
		ALL [234]	NCT01187810 NCT00104923	Terminated: drug supply Completed
		Lymphoma [356]	NCT02495415	Unknown
			NCT00288067	Terminated: drug supply
			NCT01187810	Terminated: drug supply
			NCT00104923	Completed
			NCT00589381	Completed
			NCT04234048	Not yet recruiting
			NCT01553071	Terminated: drug supply
	GT-11 [357]	ALL [238]		
AC	N-oleoylethanolamine [358]	LGL Leukemia [294]		
	SACLAC [156]	AML [156]		
	LCL204 [359]	AML [113,159]		
aSMase	Siramesine [286]	CLL [287]		
S1PR	Fingolimod (FTY720, S1PR modulator with both agonist and antagonist effects [360])	AML [185,186] T-LGL [294]		
